# Supplementary material for: Gold Nanoparticles as a Potent Radiosensitizer: A Transdisciplinary Approach from Physics to Patient
Source: Cancers (Basel). 2020 Jul 23;12(8):2021. doi: 10.3390/cancers12082021 (PMC7464732; doi:10.3390/cancers12082021)
Supplement: Supplementary file 1 [file cancers-12-02021-s001.pdf]

Supplementary Materials

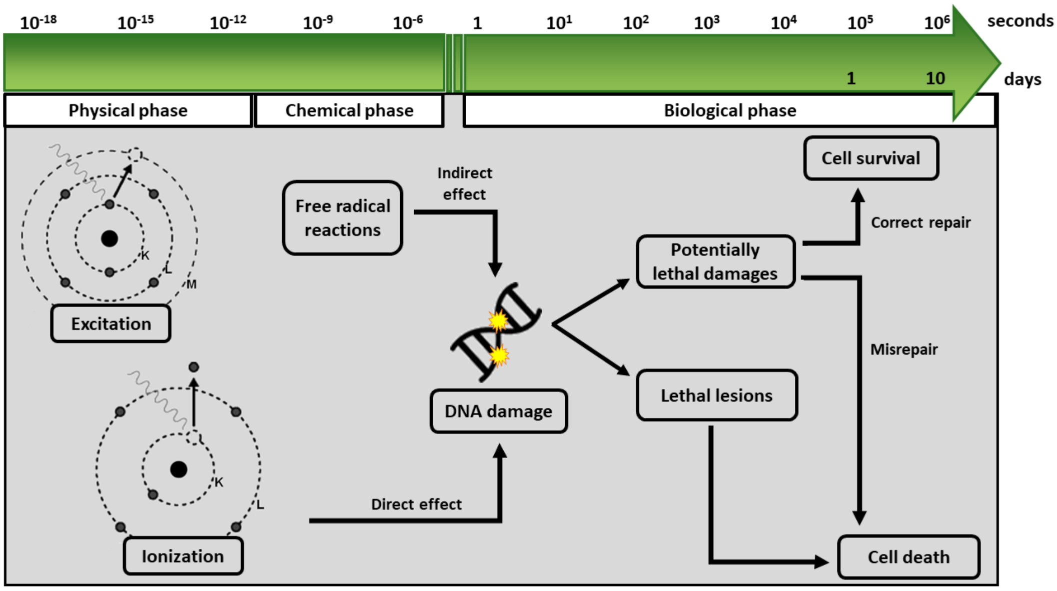

Figure S1. Time scale of post-irradiation processes and their impact on a biological system.

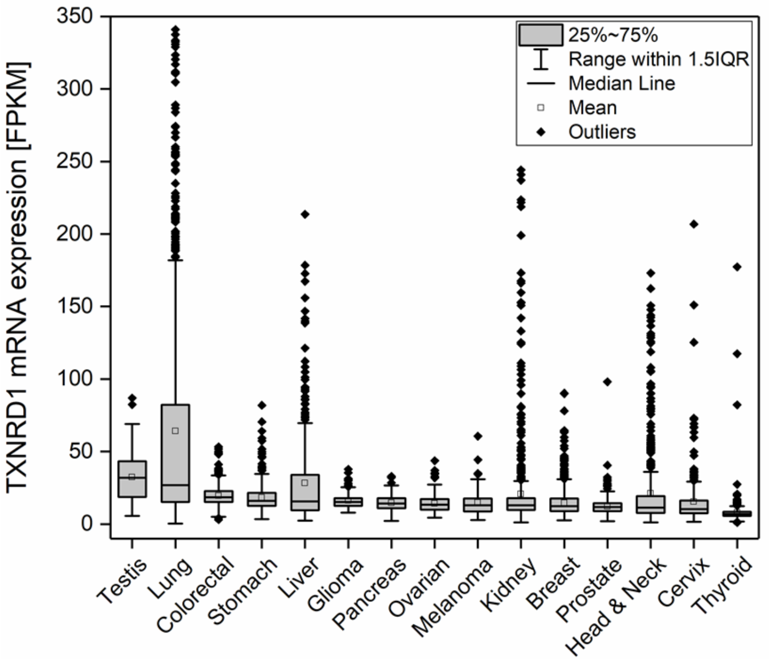

Figure S2. mRNA expression of TXNRD1 gene reported as FPKM (number fragments per kilobase of exon per million reads) according to cancer tissue. Cancer types are ranked in descending median TXNRD1 mRNA expression. Data were collected from the following TCGA datasets: breast (TCGA-BRCA, 1075 patient samples); cervix (TCGA-CESC, 291 patient samples); colorectal (TCGA-COAD & TCGA-READ, 597 patient samples); glioma (TCGA-GBM, 153 patient samples); head & neck (TCGA-HNSC, 499 patient samples); kidney (TCGA-KIRC, 877 patient samples); liver (TCGA-LIHC, 365 patient samples), lung (TCGA-LUAD & TCGA-LUSC, 994 patient samples), melanoma (TCGA-SKCM, 102 patient samples); ovarian (TCGA-OV, 373 patient samples); pancreas (TCGA-PAAD, 176 patient samples); prostate (TCGA-PRAD, 494 patient samples); stomach (TCGA-STAD, 354 patient samples); testis (TCGA-TGCT, 134 patient samples); thyroid (TCGA-THCA, 501 patient samples).
